# The Efficacy of Sodium Hypochlorite in Combination with Hyaluronic Acid as an Adjunct to Non-Surgical Periodontal Treatment: A Systematic Review

**DOI:** 10.3390/antibiotics15050428

**Published:** 2026-04-24

**Authors:** Qonita Feria, Inggrid Ratna Sari Soegiharto, Nanda Denia Astika Putri, Yohana Hutapea, Naoki Takahashi, Benso Sulijaya, Dewi Ayuningtyas

**Affiliations:** 1 Department of Periodontology, Faculty of Dentistry, Universitas Indonesia, Salemba Raya No. 4, Jakarta Pusat 10430, Indonesia; drgqonitaferia@gmail.com (Q.F.); inggridsoegiharto@gmail.com (I.R.S.S.); nandadeniaap@gmail.com (N.D.A.P.); joanvita_melody@yahoo.co.id (Y.H.); 2 Division of Periodontology, Department of Oral Health Science, Faculty of Dental Medicine, Hokkaido University, Hokkaido 060-0808, Japan; 3 Basic, Translational, and Clinical Research in Periodontology Group, Faculty of Dentistry, Universitas Indonesia, Salemba Raya No. 4, Jakarta Pusat 10430, Indonesia

**Keywords:** sodium hypochlorite, hyaluronic acid, non-surgical periodontal treatment, systematic review

## Abstract

**Objective:** The purpose of this systematic review is to evaluate the available scientific literature on the effectiveness of combining sodium hypochlorite and cross-linked hyaluronic acid (xHyA) as an adjunct to non-surgical periodontal treatment. **Materials and Methods:** Five electronic databases were searched. The study was traced using the PRISMA criteria and publications from ProQuest, Google Scholar, Springer Nature, Scopus, and PubMed. The randomized study was examined using the Cochrane Risk of Bias 2 (RoB) tool and two case series studies were reviewed using the Joanna Briggs Institute (JBI) Critical Appraisal Checklist. **Results:** The systematic review included four studies (two RCT and two case series). Across the included studies, the adjunctive use of sodium hypochlorite/amino acid gel and cross-linked hyaluronic acid (xHyA) following subgingival instrumentation was associated with improvements in clinical periodontal parameters. Probing pocket depth (PPD) reduction ranged from 1.5 to 5.8 mm, clinical attachment level (CAL) gain ranged from 1.5 to 5.3 mm, and bleeding on probing (BOP) reduction ranged from 57.5% to 65.6%. The improvements were generally more pronounced in deeper periodontal pockets. Minor variations in intervention protocols were observed among studies. **Conclusions:** The adjunctive use of sodium hypochlorite and cross-linked hyaluronic acid in non-surgical periodontal therapy may be associated with improvements in clinical periodontal parameters, including PPD, CAL, and BOP, particularly in deep pockets. However, the available evidence is limited and heterogeneous, with small sample sizes and short follow-up durations. Therefore, these findings should be interpreted with caution, and further well-designed long-term studies are required.

## 1. Introduction

Periodontitis is a chronic inflammatory disease with multiple contributing factors, primarily caused by dental biofilms that are dysbiotic [1,2,3,4]. This condition leads to an inflammatory response that damages the connective tissue connection around the teeth, causing alveolar bone resorption, which may eventually lead to tooth loss [1,2,5,6]. In periodontitis, the normal balance of healthy subgingival plaque biofilm is disrupted, compromising its role in innate defense and tissue maintenance [7]. This disruption subsequently causes excessive and uncontrolled inflammation, leading to tissue destruction [3,5,7]. The main clinical characteristics of periodontitis include the loss of periodontal tissue support, which is evidenced by clinical attachment loss and alveolar bone loss as seen on radiographs, along with gingival bleeding and the formation of periodontal pockets [1,2,6,8].

Data from the Global Burden of Disease Study indicate that severe periodontitis affected approximately 1.1 billion individuals globally in 2019, reflecting a substantial increase compared with the prevalence reported in 1990 [9]. This burden is expected to increase due to the aging population and the increased retention of natural teeth [10,11]. Moreover, periodontitis has been shown to have bidirectional relationships with systemic inflammatory conditions such as diabetes [12,13]. Therefore, the prevention, early diagnosis, and effective management of periodontitis are critically important [14].

The main objective of periodontitis treatment is to eliminate periodontal inflammation, therefore preserving, improving, and maintaining natural dentition [4,7,15]. Non-surgical periodontal therapy (NSPT) which encompasses professional mechanical plaque removal (PMPR) and subgingival instrumentation is considered the primary approach in periodontal treatment and should be performed regardless of the disease progression, as it targets the disruption of subgingival biofilm and the removal of calculus from infected root surfaces, facilitating the healing of periodontal tissues [4,11,15,16]. The main outcomes of NSPT is pocket closure, which is defined as achieving a probing pocket depth (PPD) ≤ 4 mm [17]. Although the effectiveness of non-surgical periodontal therapy is well-documented, residual periodontal pockets are often detected during re-evaluation in the course of supportive periodontal therapy (SPT) [4,10,15]. These deep residual periodontal pockets serve as reservoirs for pathogenic bacteria, contributing to persistent inflammation and an elevated risk of tooth loss [4,10]. Given the significant risk of disease progression associated with ongoing inflammation, prompt treatment of these pockets is essential for achieving long-term periodontal stability [4,10]. Residual or persistent pockets are defined as sites with a PPD of 4 mm accompanied by positive bleeding on probing (BOP) or depths of 5 mm or more at re-evaluation [18]. Recent clinical practice guidelines for managing stage II–IV periodontitis concluded that cause-related therapy focuses on reducing or eliminating the subgingival biofilm and calculus through subgingival instrumentation, potentially supplemented with adjunctive use of physical or chemical agents [10,18,19].

The use of local adjunctive antimicrobial chemo-therapeutic agents to target or suppress pathogenic periodontal microflora in areas where mechanical instrumentation is challenging has proven to be clinically relevant [7,19]. A novel idea was proposed to improve the results of non-surgical periodontal therapy by improving biofilm clearance during non-surgical therapy using sodium hypochlorite/amino acids and then applying a cross-linked hyaluronic acid (xHyA) gel [7,20,21].

Sodium hypochlorite exhibits several characteristics that support its use in periodontal therapy, including potent bactericidal effects, low toxicity at recommended concentrations, absence of color, and tooth discoloration, as well as easy accessibility and low cost [11]. Preclinical research has shown that sodium hypochlorite/amino acid gel functions as an antiseptic and antibacterial agent and that it can degrade biofilm matrices [22]. According to various studies, applying sodium hypochlorite/amino acid gel to the root surface encourages the growth and favorable survival of periodontal ligament cells [20]. Clinically, the use of this gel has proven beneficial in treating deep periodontal pockets in cases of untreated periodontitis, residual periodontal pockets, peri-implant mucositis, and peri-implantitis [22]. According to studies, using sodium hypochlorite/amino acids gel to the root surfaces encourages the growth and favorable survival of periodontal ligament cells [23].

The application of hyaluronic acid (HA) gel improves tissue repair and makes a substantial contribution to periodontal regeneration and wound healing. Additionally, hyaluronic acid has shown bacteriostatic effects against periodontal pathogens and it is beneficial in reducing bacterial contamination in surgical wounds [20,24]. Adjunctive use of topical HA in combination with NSPT has demonstrated favorable clinical outcomes, including reductions in the plaque index (PI) and the bleeding index (BI) in gingivitis patients, as well as improvements in bleeding on probing (BOP), probing depth (PD), clinical attachment level (CAL), and the subgingival bacterial profile compared with NSPT alone [25]. According to several systematic reviews and meta-analyses, the application of hyaluronic acid gel shows promising potential as an adjunct to NSPT [24,26,27]. Histological data from animal studies have demonstrated that the use of cross-linked hyaluronic acid as an adjunctive treatment led to significant regeneration of periodontal tissues in comparison to surgical controls when treating furcation defects, gingival recessions, or intrabony defects [22].

Sodium hypochlorite acts as a cleaning agent by dissolving necrotic tissue, modifying biofilm matrices, specifically targeting Gram-negative bacteria linked to periodontitis, and softening calculus. After the application of sodium hypochlorite, cross-linked hyaluronic acid gel is applied to sites to enhance wound healing, significantly stimulating blood clot formation and promoting periodontal wound healing/regeneration [22,28]. High-molecular-weight hyaluronic acid, produced by hyaluronan synthase enzymes in periodontal tissues, gingiva, periodontal ligaments, and alveolar bone, is extensively degraded in chronically inflamed tissue, which allows it to regulate the inflammatory response [29]. Furthermore, throughout the healing process, HA prevents the breakdown of extracellular matrix by serine proteinases secreted from inflammatory cells, which indirectly reduces inflammation and stabilizes the granulation tissue [29]. Many cells in the periodontium express hyaluronic acid, which is a significant part of the extracellular matrix. Cementoblasts and periodontal ligament cells both display its primary receptor, CD44, a cell surface protein that is crucial for periodontal ligament mineralization. This suggests that HA may play a part in periodontal regeneration by binding to CD44 in these cells [29].

Therefore, the purpose of this systematic review is to evaluate the available scientific literature of the possible effectiveness of combining sodium hypochlorite and cross-linked hyaluronic acid (xHyA) as an adjunct to non-surgical periodontal treatment.

## 2. Materials and Methods

This systematic review has been registered with PROSPERO, CRD42024618961. The study question in this systematic review was “Is there any added benefit (O) in the application of sodium hypochlorite in combination with hyaluronic acid as an adjunct (I) compared to non-surgical periodontal treatment without adjunctive therapy (C) in periodontitis patients (P)?”. This research question is translated using PICO which consists of Population, Intervention, Comparison, and Outcome. In Table 1, a PICO description table is shown.

### 2.1. Search Strategy

Relevant studies were retrieved through an electronic search of selected databases restricted to 5 years of publication. The most recent search was carried out in December 2024. Four independent reviewers (Q.F., I.R., N.D. and Y.H.) conducted the search across five databases, including ProQuest, Google Scholar, Springer Nature, Scopus, and PubMed. The search strategy utilized combinations of the following keywords: ((periodontitis) AND ((periodontal treatment) OR (non-surgical periodontal treatment)) AND (combination) AND (sodium hypochlorite) AND (hyaluronic acid)). Only studies published in English were included. Keywords from the PICO analysis may be grouped by combining non-surgical periodontal therapy, sodium hypochlorite, hyaluronic acid, periodontitis, and periodontal treatment.

### 2.2. Eligibility Criteria

The inclusion criteria were as follows: (1) randomized clinical trials and case series evaluating the administration of sodium hypochlorite in combination with hyaluronic acid as an adjunct to non-surgical periodontal treatment; (2) studies reporting primary clinical outcomes including probing pocket depth, clinical attachment level, bleeding on probing, and the plaque index; (3) studies including participants diagnosed with periodontitis according to the 2017 World Workshop on the Classification of Periodontal and Peri-Implant Diseases and Conditions; and (4) studies with a follow-up period ranging from 3 to 9 months. Due to the limited number of randomized clinical trials available on this topic, case series were also included in order to provide a broader overview of the currently available clinical evidence. This inclusion allowed the review to capture relevant clinical outcomes reported in the literature.

### 2.3. Study Selection and Data Extraction

The titles and abstracts of the identified publications were first examined to determine whether they were relevant to the objective of this systematic review. Studies that were duplicated or did not meet the scope of the review were excluded at this stage. The remaining articles then underwent a full text evaluation based on the predefined inclusion criteria, and only those that satisfied the eligibility requirements were included in the final analysis. Furthermore, the reference lists of the included articles were manually checked to identify any additional relevant studies that might have been missed during the initial search.

The processes of study selection, data extraction, and the risk of bias assessment were carried out independently by two reviewers (Q.F. and N.D.). Any discrepancies between the two reviewers were discussed, and if consensus could not be reached, a third reviewer (I.R.) was consulted to make the final decision.

## 3. Results

**Figure 1:**
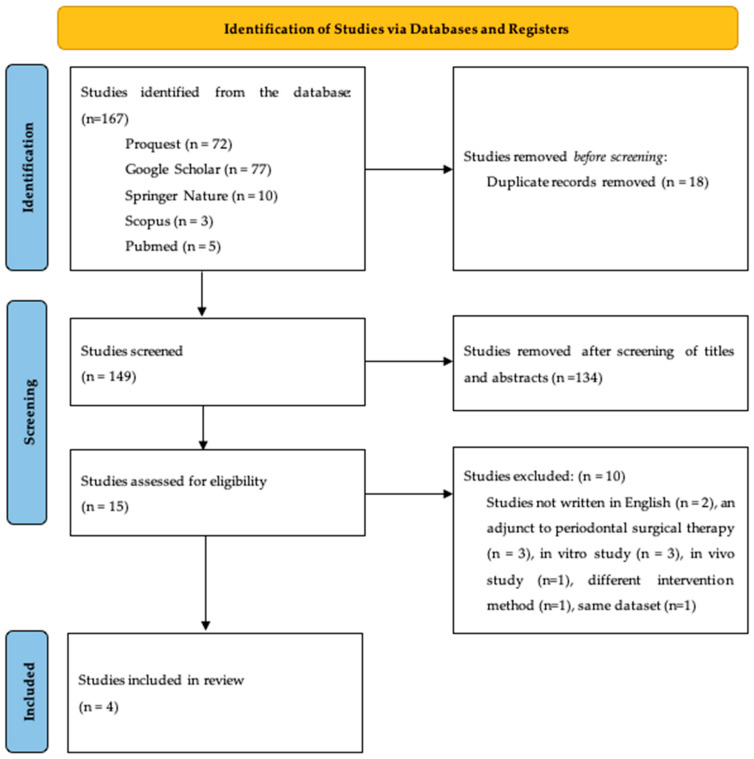
PRISMA flow diagram.

Research identification was conducted in accordance with the PRISMA (Preferred Reporting Items for Systematic Reviews and Meta-Analyses) guidelines, and the flowchart is presented in Figure 1. The search across five electronic databases using the specified keywords yielded a total of 167 studies, including 72 from ProQuest, 77 from Google Scholar, 10 from Springer Nature, 3 from Scopus, and 5 from PubMed (Table 2). All records were imported into Microsoft Excel, and 18 duplicate studies were identified and removed. Title and abstract screening were then performed on the remaining 149 studies, of which 134 were excluded for not meeting the predefined inclusion and exclusion criteria. Consequently, 15 studies were selected for full text assessment. Following full text review, 10 studies were excluded: two were not published in English, three evaluated adjunctive treatment in conjunction with periodontal surgical therapy, three were in vitro studies, one was an in vivo study, one used a different intervention method, and one reported data derived from the same dataset as another included study.

### 3.1. Characteristics of the Included Studies

Among the four included studies, two were randomized controlled trials (RCTs) and two were case series. Additional study characteristics are summarized in Table 3. The RCTs demonstrated comparable methodologies, typically involving two groups: a control group, in which subgingival instrumentation was performed without adjunctive agents, and an intervention group, in which subgingival instrumentation was combined with locally administered sodium hypochlorite and hyaluronic acid gel. All studies assessed treatment outcomes using clinical periodontal parameters measured intraorally, while one study additionally reported microbiological outcomes alongside the clinical parameters. The studies showed some variations in intervention protocols, follow-up duration, and participant characteristics. Nevertheless, all studies evaluated the adjunctive use of sodium hypochlorite and cross-linked hyaluronic acid during non-surgical periodontal therapy, particularly subgingival debridement. The most commonly reported clinical outcomes included periodontal probing depth (PPD), bleeding on probing (BOP), and clinical attachment level (CAL).

### 3.2. Characteristics of Interventions

All participants were assessed and underwent full-mouth mechanical debridement with manual instruments and/or ultrasonic devices. Adjunctive to subgingival debridement, sodium hypochlorite/amino acid gel, was instilled into pockets with a probing depth of 4 mm or more. Three studies applied the gel for 60 s, while other studies applied the gel for 30–45 s before subgingival instrumentation. The application of sodium hypochlorite was repeated until instrumentation was considered sufficient. Without rinsing the sodium hypochlorite gel with water or saline, the application of cross-linked hyaluronic acid was done by inserting the gel into the pockets. After undergoing the intervention, the participants were given individual oral hygiene instructions. One study by Benyei et al. [10] reapplied cross-linked hyaluronic acid gel 7 days post-treatment, while other studies did not. The participants were instructed to commit to a 3- to 9-month evaluation period.

### 3.3. Characteristics of Outcome Measures

Several clinical periodontal parameters were observed to evaluate the efficacy of combined sodium hypochlorite and hyaluronic acid as an adjunct to subgingival instrumentation, namely periodontal probing depth (PPD), clinical attachment loss (CAL), and bleeding on probing (BOP). Several studies also evaluated gingival recession and plaque index as their outcomes. The evaluation of periodontal healing was done by comparing the measurements of the clinical parameters at baseline and at follow-ups.

### 3.4. Characteristics of Outcomes

All four studies included in this review showed a significant reduction in PPD and BOP values with a significant gain in CAL with the use of combined sodium hypochlorite and hyaluronic acid (Table 4). In the randomized control trials, significant PPD reduction and CAL gain was observed in both control and intervention groups, but favoring the intervention treatment group; while BOP reduction was also found statistically significant in both groups, no statistical difference was observed between groups in the study performed by Benyei et al. [10] In the case series, a significant PPD reduction and CAL gain were observed in both studies. Both studies also revealed a BP reduction when comparing baseline assessments, as well as at both follow-up evaluations.

Studies by Benyei et al. [10], Ramanauskaite et al. [7,20,22], and Diehl et al. [21] have shown that deeper pockets applied with sodium hypochlorite and hyaluronic acid tend to benefit the most when compared to shallower pockets. The study by Ramanauskaite et al. [7,20,22] also revealed that there was a significant improvement in the plaque index after the intervention.

### 3.5. Risk of Bias Assessment

**Figure 2:**
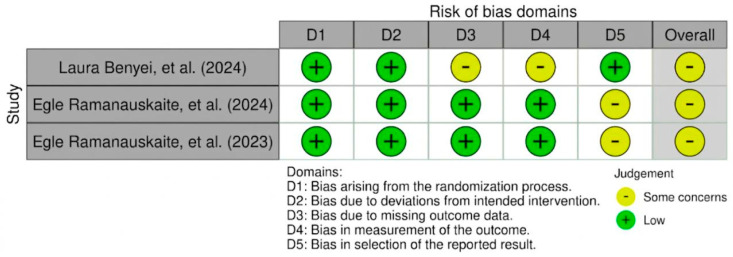
The results of the risk of bias assessment for randomized clinical trials [7,10,22].

**Figure 3:**
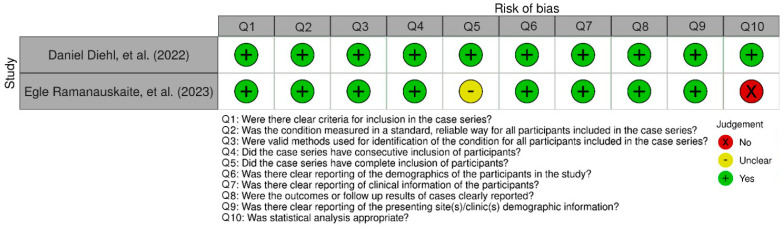
The results of the risk of bias assessment for case series [20,21].

The evaluation of the risk of bias (RoB) was conducted independently by four authors (Q.F., I.R., N.P. and Y.H.), with the supervisor (B.S. and D.A.) providing input for final decisions; any disagreements were resolved through discussion until consensus was reached. The randomized study was reviewed using the Cochrane Risk of Bias Version 2 (RoB) tool. Three studies were assessed using this tool and the assessments are summarized in Figure 2, where one study seems to indicate some concerns regarding the risk of bias. Two case series used in this study were reviewed using the Joanna Briggs Institute Critical Appraisal Checklist and the assessment is shown in Figure 3.

## 4. Discussion

This systematic review consisted of two RCTs and two case series, in which the risk of was needed to be assessed to evaluate the quality of each study methodologically. The risk of bias (RoB) assessment indicated that the included randomized controlled trials (RCTs) were generally of acceptable methodological quality, with most domains rated as low-risk. Proper randomization procedures and allocation concealment were reported across the RCTs, minimizing selection bias. However, some concerns were identified in specific domains. In the study performed by Laura Benyei, a small number of participant withdrawals occurred during the follow-up, and the reasons for dropout were not clearly reported. Although the number of missing cases was limited and balanced between groups, the lack of detailed information regarding the causes of withdrawal resulted in some concerns related to missing outcome data. Additionally, some concerns regarding the absence of clear blinding of the outcome assessor were raised, which may have introduced detection bias, particularly for clinically measured periodontal parameters. Similarly, another included RCT was judged to have some concerns in the overall risk of bias assessment due to incomplete reporting of one of the pre-specified outcomes, which may limit the comprehensiveness of the reported findings. For the included case series, an inherently higher risk of bias must be acknowledged due to the absence of randomization, control groups, and blinding. These limitations reduce the strength of causal inference and increase susceptibility to confounding and selection bias. Nevertheless, the case series provided supportive clinical observations that were consistent with the findings of the RCTs, particularly regarding improvements in key periodontal parameters.

Non-surgical periodontal treatment is a method used to treat periodontitis with the aim of eliminating bacterial agents and reducing inflammation through a conventional approach [30]. According to the European Federation of Periodontology (EFP) Treatment Guidelines, after the confirmation of the diagnosis, there are two steps that must be followed before any need of surgical approach. The first step is “behavior and risk factor modification” including supragingival professional mechanical plaque removal (PMPR), oral hygiene instruction, and control of risk factors; the second step is “cause-related therapy” that is aimed at controlling the subgingival biofilm and calculus through subgingival instrumentation and that may be supplemented with the use of adjunctive therapeutic agents [18]. However, the complete removal of subgingival biofilm and calculus deposits using conventional non-surgical periodontal therapy remains challenging, particularly in deeper periodontal pockets, where access is limited. While surgical approaches such as open flap debridement may improve visibility and facilitate more effective calculus removal, they are associated with potential drawbacks, including postoperative discomfort, increased risk of complications, longer healing periods, and gingival recession leading to root surface exposure [31]. These limitations may reduce patient acceptance of surgical interventions, thereby emphasizing the need for adjunctive strategies that can enhance the effectiveness of non-surgical periodontal therapy. In this review, we selected adjunctive agents containing sodium hypochlorite gel and cross-linked hyaluronic acid for review due to recent studies indicating their potential for enhancing clinical periodontal outcomes when used alongside non-surgical periodontal therapy.

**Figure 4:**
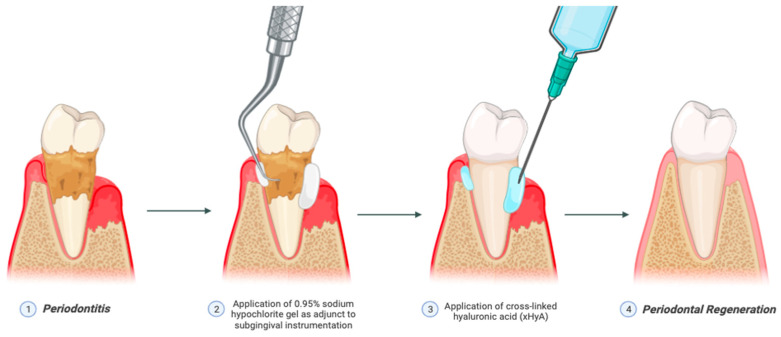
Application of sodium hypochlorite gel and cross-linked hyaluronic acid in periodontal treatment. (**1**) The impact of dental plaque and biofilm dysbiosis is the destruction of periodontal structures. (**2**) Adjunctive use of 0.95% sodium hypochlorite gel in persistent periodontal pockets to subgingival instrumentation. (**3**) Cross-linked hyaluronic acid (xHyA) was administered after sufficient subgingival instrumentation. (**4**) Synergistic therapeutic benefits by combining these two adjuncts promotes periodontal regeneration. Illustrated/modified by F.Q.

Recently, the formulation of sodium hypochlorite gel buffered with leucine, lysine, and glutamic acid, which are categorized as amino acids, has been utilized as an adjunct to subgingival instrumentation and re-instrumentation in supportive periodontal therapy (Figure 4) [11,32]. This formulation consists of a diluted sodium hypochlorite solution and a viscous amino acid solution, which are mixed together to form a viscous alkaline amino acid solution [23]. These active ingredients contained in the gel create chloramines, which have a strong antimicrobial effect, and which can soften the calculus and reduce friction during instrumentation, thus increasing the effect of SRP [33]. In an in vitro study, the use of 0.3% sodium hypochlorite showed a significant reduction in the total biofilm mass and reduced the viability of the bacteria [23]. Prior histological studies on dogs treated with sodium hypochlorite and hyaluronic acid showed more bone, cementum, and connective attachment formation in the treatment group [28]. Additionally, a recent randomized controlled trial reported that the adjunctive application of sodium hypochlorite alongside minimally invasive non-surgical therapy (MINST) in untreated periodontitis patients with deep periodontal pockets led to significant improvements in probing depth (PD) and clinical attachment level (CAL), while no significant effect on gingival recession was observed [34]. Sodium hypochlorite primarily facilitates biofilm disruption and chemical debridement, enhancing root surface decontamination, whereas hyaluronic acid contributes to the promotion of wound healing as it acts as an anti-inflammatory agent, enhances clot formation, induces angiogenesis, promotes osteogenesis, and has an important role in cell differentiation, adhesion, and migration [33,35]. A systematic review by Mehta et al. suggests reduction in gingival recession, gains in CAL, and complete root coverage after local application of hyaluronic acid. It also appears to have a major role in both mineralized and non-mineralized periodontal tissues (alveolar bone, cementum, and periodontal ligament) [36]. The improved clinical outcomes observed with the adjunctive protocol may be attributed to the complementary mechanisms of sodium hypochlorite and cross-linked hyaluronic acid, whose synergistic effects create a more favorable microenvironment for periodontal tissue repair and enhance the overall therapeutic outcome beyond mechanical instrumentation alone.

This review included four studies that collectively highlight the potential benefits of combining sodium hypochlorite gel and cross-linked hyaluronic acid as an adjunctive to non-surgical periodontal treatment. Mechanical debridement, performed manually or with a combination of manual and ultrasonic scalers, was used to remove subgingival biofilm and calculus in all the included studies. This process was preceded by repeated applications of sodium hypochlorite gel and followed by the application of hyaluronic acid. In all studies included in this review, both randomized controlled trials and case series showed significant improvement in clinical parameters, particularly reductions in PPD and gain in CAL. The combination of sodium hypochlorite gel and cross-linked hyaluronic acid as an adjunct to non-surgical periodontal therapy has been observed to improve outcomes compared to subgingival instrumentation alone. It is also evident from multiple studies that deep periodontal pockets consistently benefit the most from adjunctive sodium hypochlorite and hyaluronic acid therapy, regardless of variations in the definition of “deep pockets” between studies [7,10,20,22]. While one study defined deep pockets as sites with a PPD of 8 mm or more [10], other studies defined them as sites with a PPD of 7 mm or more [7,20,22]. Despite these differences, there is a similar pattern across studies: when treated with the adjunctive protocol, deep pockets revealed noticeably greater clinical improvements when compared to moderate or shallow pockets [7,10,20,22]. This finding is in line with a recent systematic review which also found that shallow sites (4–6 mm) had a mean reduction in PPD of 1.5 mm; however, deeper sites (≥7 mm) had a higher mean reduction in PPD at 2.6 mm after undergoing subgingival instrumentation that could be expected at 6 to 8 months post-treatment [37]. The greater responsiveness of deeper pockets may be explained by their higher microbial burden and the limited accessibility of these sites, where adjunctive agents may provide additional benefits by enhancing biofilm disruption and supporting tissue healing [11].

Beyond reductions in probing depth, several studies also reported outcomes related to pocket closure, which may provide a more clinically meaningful indicator of treatment success. In the study conducted by Ramanauskaite et al., a significantly higher proportion of shallow pockets (1–3 mm) was observed in the test group compared to the control group at both 3 and 6 months, indicating superior pocket closure following adjunctive therapy [20]. Similarly, Benyei et al. reported a pocket closure rate of 94% in severely deep pockets (≥8 mm) in the test group, highlighting the potential effectiveness of the adjunctive protocol in challenging clinical scenarios [10]. In contrast, Diehl et al. reported a low pocket closure rate of 25.25% in single-rooted teeth, particularly given the relatively short application time of sodium hypochlorite (30–45 s), which may have limited its biofilm penetration and overall therapeutic effect [21]. Although these findings support the potential benefits of adjunctive therapy, the clinical relevance of the observed superiority should be interpreted with caution. Furthermore, while probing depth reduction is an important indicator of treatment response, it should not be considered as the sole endpoint of periodontal therapy, as true periodontal stability depends on the resolution of inflammation and the achievement of shallow, non-bleeding pockets. According to current treatment guidelines, if the endpoints of therapy—defined as the absence of periodontal pockets > 4 mm with bleeding on probing or the presence of deep pockets (≥6 mm)—are not achieved, progression to step 3 (surgical therapy) should be considered [18].

However, not all clinical outcomes exhibited consistent intergroup differences across the studies. The randomized clinical study conducted by Laura Benyei et al. revealed varying statistical outcomes regarding mean BOP reduction when compared to other included studies. Though mean BOP reduction was observed in both test and control groups, no statistically significant differences were found between groups. This result differs from findings in other studies, where it was observed that the combination of adjuvants consistently resulted in a more significant reduction in BOP than subgingival instrumentation alone [7,20,22]. The absence of notable differences in BOP frequency in both groups observed from baseline to the last re-evaluation in this study may be related to an external factor—smoking—which has been observed to suppress gingival bleeding upon probing [38]. Unlike other studies, the study conducted by Laura Benyei et al. included smokers—30% of the participants from both groups were active smokers. In a recent study, it has been confirmed that smokers had less gingival bleeding upon probing to the bottom of the pocket/sulcus than non-smokers [39]. Evidence suggests that smoking could suppress gingival bleeding due to thermally induced nerve damage, which in turn reduces angiogenesis and modifies the gingival inflammatory response [24,39].

Minor variations in the duration and frequency of sodium hypochlorite and cross-linked hyaluronic acid application was also observed between studies, although the overall methodology of the intervention remained consistent, particularly regarding the duration of sodium hypochlorite application and the reapplication of hyaluronic acid. The application duration of sodium hypochlorite gel varied between studies, ranging from 30 s to 60 s, with repeated applications performed until decontamination was considered sufficient. While these variations did not appear to significantly influence clinical outcomes, suggesting that application time may not critically affect therapeutic efficacy, other evidence indicates that duration may still play a role in this process. For instance, the limited effect of 0.95% sodium hypochlorite buffered with amino acids reported by Iorio-Siciliano et al. may be attributed to the shorter application time of 30 s [34]. In contrast, a longer application time of 60 s has been suggested to enhance biofilm penetration and facilitate its removal, thereby potentially improving treatment effectiveness [20,40]. However, it is noteworthy that an in vitro study reported a 35% decrease in viability of human gingival fibroblast when exposed to 0.1% sodium hypochlorite solution for 5 min [23].

The reapplication of cross-linked hyaluronic acid 7 days post-treatment was another procedural difference observed in the study conducted by Laura Benyei et al. While the reapplication was most likely proposed to prolong the therapeutic benefits of the adjuvant by maintaining its presence in the periodontal pocket, it is possible that the lack of significant differences in outcomes between reapplication and no reapplication was associated with the slow degradation profile of cross-linked hyaluronic acid, which can maintain a longer presence of 4–6 weeks [7,41]. This is further supported by evidence from several studies showing statistically significant reductions in PPD and gains in CAL between follow-ups, even without reapplication of the adjuvant, emphasizing its prolonged stability and sustained therapeutic effects. This inherent stability may reduce the need for repeated reapplications, which could explain the lack of consistent clinical improvement directly attributed to the reapplication of cross-linked hyaluronic acid.

From an economic perspective, the clinical relevance of this improvement should be carefully evaluated in relation to the associated increase in treatment costs. Although an average reduction of approximately 1 mm may appear relatively small, it may still be considered clinically meaningful in deep periodontal pocket (>6 mm) where even small improvements can enhance plaque control accessibility. However, adjunctive therapy often involves additional financial burden, which may not be justified in all cases. Therefore, its use should not be considered routine but rather for selected clinical situations, such as deep or complex defects where the potential benefits may outweigh the cost.

Another important finding was that the combination of sodium hypochlorite and cross-linked hyaluronic acid consistently resulted in uneventful healing of soft tissues across the studies included in this review. There were no reports of adverse reactions, discomfort, or complications following the application of the combined adjuvants, which further reinforces their safety of use. However, despite the consistent positive findings, it is important to consider that the observed clinical improvements may not be solely attributed to the adjunctive intervention. This finding is particularly significant, as the potential clinical relevance of the local application of sodium hypochlorite and cross-linked hyaluronic acid in conjunction with mechanical subgingival instrumentation remains limited at present.

All included studies involved participants with a high level of oral hygiene, as patients had undergone the initial periodontal therapy and received continuous oral hygiene instructions, which may have independently contributed to the observed clinical improvements. Furthermore, the differences in study design, particularly the inclusion of residual versus untreated periodontal pockets, may have influenced the magnitude of clinical outcomes, potentially leading to an overestimation of the adjunctive effect.

**Figure 5:**
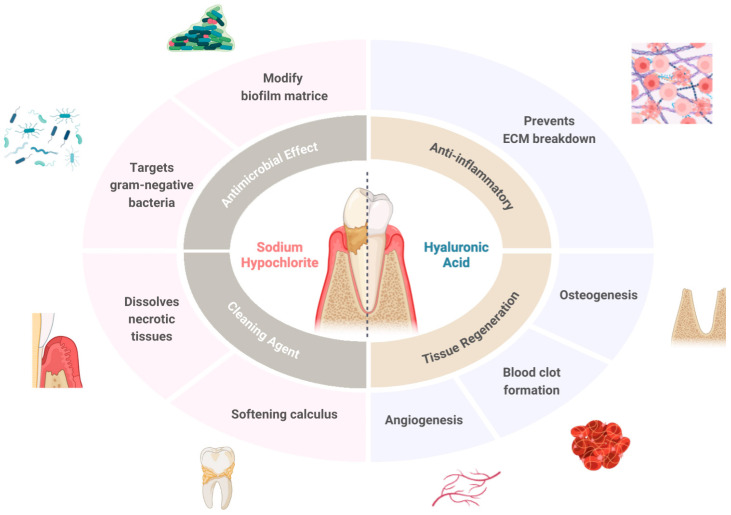
Properties of sodium hypochlorite and hyaluronic acid in periodontal therapy. Sodium hypochlorite acts as a cleaning agent with antimicrobial effects by softening calculus, dissolving necrotic tissue, and targeting Gram-negative periodontal pathogens. Meanwhile, hyaluronic acid promotes periodontal tissue regeneration by promoting angiogenesis, osteogenesis, and blood clot formation, while also functioning as an anti-inflammatory agent for preventing the extracellular matrix from breaking down and ensuring periodontal healing. Illustrated/modified by F.Q.

The effectiveness of non-surgical periodontal therapy in the management of periodontitis has been widely reported in the literature, and NSPT remains the gold standard for periodontal treatment. Nevertheless, there is a continuing need for adjunctive approaches that can enhance the effects of NSPT without introducing adverse effects. Low-concentration sodium hypochlorite buffered with amino acids has been proposed as a potential option with such properties and has therefore been the focus of considerable research (Figure 5). This review highlights the effectiveness of sodium hypochlorite gel combined with cross-linked hyaluronic acid as an adjunctive agent following NSPT. However, our study has several limitations. First, the included studies comprised both randomized controlled trials and case series, which differ in methodological rigor and may complicate the interpretation and synthesis of the overall findings. Second, the studies included relatively small sample sizes, which may influence the reliability and generalizability of the results. Third, the follow-up periods were relatively short, with a maximum duration of 6 months in most studies and only one study extending to 9 months. To obtain more definitive results and better assess long-term effectiveness, future studies should incorporate follow-up periods of at least 1 year. Fourth, variability in study design may have influenced the reported clinical outcomes, as some studies evaluated residual pockets following re-evaluation after initial therapy, while others assessed untreated periodontal pockets, which are generally known to demonstrate greater clinical improvement [11]. Fifth, all study participants demonstrated a relatively high level of oral hygiene, as they had undergone the initial therapy and received continuous oral hygiene instructions, including proper toothbrushing techniques and the use of interdental cleaning aids, which may have contributed to the observed clinical improvements independent of the adjunctive interventions. Finally, one of the included studies presented some concerns regarding the risk of bias, as it was conducted as a single-center investigation with a single operator, potentially limiting its robustness. Moving forward, further research is needed to standardize the optimal concentration, application protocols, and follow-up duration for the local use of these adjunctive agents.

## 5. Conclusions

This systematic review highlights the clinical efficacy of sodium hypochlorite and cross-linked hyaluronic acid’s combined application as an adjunct in non-surgical periodontal therapy, specifically subgingival debridement. These combined adjuncts show additional benefits in clinical periodontal parameters with no report on any side effects or complications. Across all included studies, the use of sodium hypochlorite and cross-linked hyaluronic acid at persistent periodontal sites showed generally reported improvements in clinical parameters such as periodontal probing depth (PPD) reduction, mean bleeding on probing (BOP) reduction, and clinical attachment level (CAL) gain, particularly in deep periodontal pockets. The potential benefits observed may be related to antimicrobial properties and cleaning ability of sodium hypochlorite, coupled with hyaluronic acid anti-inflammatory and regenerative properties, creating a synergistic therapeutic benefit and thereby increasing periodontal healing.

Minor variations in application protocols were observed between studies, though the overall methodology demonstrated consistent biocompatibility. These findings suggest that the adjunctive use of sodium hypochlorite and hyaluronic acid while undergoing subgingival instrumentation offers a promising approach to improving outcomes in non-surgical periodontal therapy when compared to subgingival instrumentation alone, with the greatest clinical benefits appearing to occur in deeper periodontal pockets. Nevertheless, this should be interpreted with caution, as small sample sizes, brief follow-up times, and methodological inconsistencies highlight the need for larger, longer-term research to validate these results and establish the best practices for the clinical use of these combined adjuncts to periodontal treatment.

## Figures and Tables

**Table 1:** PICO description.

Population (P)	Intervention (I)	Comparison (C)	Outcome (O)
Systemically healthy adult patients with stage II–IV periodontitis	NSPT with adjunctive local administration of sodium hypochlorite gel combined with hyaluronic acid.	Subgingival instrumentation alone.	Primary outcome: bleeding on probing (BOP), periodontal probing depth (PPD), clinical attachment loss (CAL).

**Table 2:** Database and keywords.

Database	Keywords
ProQuest https://www.proquest.com/ (accessed on 2 December 2024)	((periodontitis) AND ((periodontal treatment) OR (non-surgical periodontal treatment)) AND (combination) AND (sodium hypochlorite) AND (hyaluronic acid)); source type: scholarly journals; publication date: last 5 years; search language: English.
Google Scholar https://scholar.google.com/ (accessed on 2 December 2024)	(“periodontitis”) AND (“periodontal treatment” OR “non-surgical periodontal treatment”) AND (“combination”) AND (“sodium hypochlorite”) AND (“hyaluronic acid”); publication date: last 5 years.
Springer Nature https://link.springer.com/ (accessed on 3 December 2024)	((periodontitis) AND ((periodontal treatment) OR (non-surgical periodontal treatment)) AND (combination) AND (sodium hypochlorite) AND (hyaluronic acid)); date published: 2019–2024; languages: English.
Scopus https://www.scopus.com/ (accessed on 3 December 2024)	periodontitis AND periodontal treatment OR non-surgical periodontal treatment AND combination AND sodium hypochlorite AND hyaluronic acid.
PubMed https://pubmed.ncbi.nlm.nih.gov/ (accessed on 2 December 2024)	((periodontitis) AND ((periodontal treatment) OR (non-surgical periodontal treatment)) AND (combination) AND (sodium hypochlorite) AND (hyaluronic acid)); publication date: last 5 years; text availability: full text.

**Table 3:** Characteristics of the selected studies.

Leading Author, Year	Country of the Study	Study Design	Inclusion Criteria	Exclusion Criteria	Number of Participants
Ramanauskaite et al. (2023, 2024) [7,22]	Lithuania	RCT	Adult males and females aged ≥18 years. Generalized periodontitis stage II–III, grades A/B. Good general health. Presence of at least 20 teeth. Absence of removable dentures. Willing to consent and complete the 6-month study.	Patients already included in other RCTs. Smokers. Periodontal treatment during the last 12 months. Antibiotic treatment 3 months prior to the start of the trial. Antibiotic prophylaxis before dental treatment. Ongoing medication that may affect periodontitis. Pregnancy/lactating. Allergies to sodium hypochlorite.	Randomized (48 patients). Test group (24 patients). Control group (24 patients).
Benyei et al. (2024) [10]	Germany	RCT	Systematically healthy adults previously diagnosed with periodontitis, showing residual periodontal pockets (PPD ≥ 5 mm or 4–5 mm with BOP) at the first re-evaluation after completing step 1–2 periodontal therapy, initially presenting with untreated stage III or IV periodontitis.	Uncontrolled T2DM with HbA1c scores > 7.5%. Other chronic conditions such as rheumatoid arthritis or pregnancy and lactating.	Initial: randomized (54 patients); rest (27 patients), control (27 patients). Final: completed the study (50 patients); test (27 patients), control (27 patients).
Diehl et al. (2022) [21]	Germany	Case series	Patients with persistent deep pockets depths (≥5 mm with positive BOP) who have already undergone SPT re-evaluation at least twice. Sites had never been subjected to any surgical intervention.		29 patients.
Ramanauskaite et al. (2023)[20]	Lithuania	Case series	Systematically healthy patients. Stage II–III periodontitis (at least one pocket in each quadrant with PD ≥ 5 mm. Radiographic bone loss (>2 mm from CEJ). A minimum of 20 teeth (wisdom teeth excluded). No removable prosthesis.	Patients already included in other studies. Smokers. Periodontal treatment during the last 12 months. Antibiotic treatment 6 months prior to the start of the trial. Antibiotic prophylaxis before dental treatment. Ongoing medication that may affect periodontitis. Pregnancy/lactating.	21 patients.

**Table 4:** Characteristics of outcome measures, interventions, and outcomes.

Leading Author, Year	Study Parameters/Outcomes	Control Details	Intervention Details	Follow-Up Time	Outcome
Ramanauskaite et al. (2023, 2024) [7,22]	PD, CAL, BOP, PI, REC.	Full-mouth SRP performed with ultrasonic and hand instruments, followed by polishing using low-abrasive paste.	Full-mouth SRP performed with ultrasonic and hand instruments, followed by polishing using low-abrasive paste. In all pockets with PD ≥ 4 mm, SI was done by applying a sodium hypochlorite/amino acid gel for 60 s prior to SI. Application of sodium hypochlorite/amino acid gel was repeated until SI was considered sufficient. Application of cross-linked hyaluronic acid gel was done following SI.	3 months, 6 months	Both groups demonstrated improvements in clinical parameters at 3 and 6 months compared to baseline, including PD, CAL, BOP, and PI; however, the test group showed significantly greater improvements compared to the control group (*p* < 0.001). Both moderate (4–6 mm) and deep (≥7 mm) pockets were reduced in both groups, with statistically significant differences observed between groups for both categories (*p* < 0.001).
Benyei et al. (2024) [10]	PPD, CAL, BOP, GR.	Subgingival instrumentation by using Gracey curettes, ultrasonic instruments, and glycine powder air polishing.	Subgingival instrumentation by using Gracey curettes, ultrasonic instruments, and glycine powder air polishing. Residual pocket sites were treated with sodium hypochlorite cleaning gel for 30 s prior to SI. Application of sodium hypochlorite gel was repeated twice. Sites were then treated with 0.2–0.3 mL of xHyA up to the gingival margin. Reapplication of xHyA (0.2–0.3 mL) was done 7 days after.	3 months, 9 months	Overall, both groups demonstrated improvements in clinical parameters, including CAL, PPD, and GR; however, the test group showed significantly greater improvements compared to the control group (*p* < 0.001), with the exception of BOP, which did not differ significantly between groups at any time point, neither at baseline (*p* = 0.796), 3-month (*p* = 0.175), nor 9-month evaluation (*p* = 0.339). Pocket closure rate in severely deep pockets (≥8 mm) in the test group was 94%.
Diehl et al. (2022) [21]	PPD, CAL, BOP.	-	Subgingival instrumentation following supragingival mechanical debridement, performed by calibrated operators using Gracey curettes. Sodium hypochlorite cleaning gel was applied subgingivally for 30–45 s and repeated until adequate instrumentation (smooth root surface) was achieved. Subsequently, 0.3 mL of cross-linked hyaluronic acid (xHyA) was applied into the subgingival pocket (flapless). Reapplication of xHyA (0.3 mL) was performed after 7 days.	5–6 months, 12 months	Significant reductions in PPD (−2.04 mm) and gains in CAL (+2.02 mm) at 6 months compared to baseline, with no increase in gingival recession. BOP decreased from 97.6% to 40.1%. CAL gain was significant in both furcation-involved teeth (1.5 mm; *p* = 0.0195) and single-rooted teeth (2.04 mm; *p* < 0.001). Pocket closure was achieved in 25.25% of sites in single-rooted teeth.
Ramanauskaite et al. (2023) [20]	PD, CAL, BOP, REC.	-	Full-mouth SRP performed with ultrasonic and hand instruments, followed by polishing using low-abrasive paste. In all pockets with PD ≥ 4 mm, SI was done with applying a sodium hypochlorite/amino acid gel for 60 s prior to SI. Application of sodium hypochlorite/amino acid gel was repeated until SI was considered sufficient (2–3 times). Application of cross-linked hyaluronic acid gel was done following SI.	3 months, 6 months	Statistically significant reduction in PD was observed at 3 months (2.6 ± 0.4 mm) and 6 months (2.9 ± 0.4 mm) compared to baseline (*p* < 0.001). Similarly, a statistically significant reduction in mean BOP was also observed both 3 months (54.9 ± 19.6%) and 6 months (65.6 ± 16.4%) following the treatment (*p* < 0.001). A statistically significant CAL gain was obtained at 3 months (2.3 ± 0.5 mm) and 6 months (2.6 ± 0.5 mm) compared to baseline (*p* < 0.001).

PD: probing depth; PPD: periodontal pocket depth; CAL: clinical attachment loss; BOP: bleeding on probing; REC, GR: gingival recession; SRP: scaling and root planning; xHyA: cross-linked hyaluronic acid.

## Data Availability

The original contributions presented in this study are included in the article. Further inquiries can be directed to the corresponding authors.
